# Examining symptom trajectories that predict worse outcomes in post-CABG patients

**DOI:** 10.1177/1474515118809906

**Published:** 2018-10-31

**Authors:** Ming-Fen Tsai, Shiow-Luan Tsay, Debra Moser, Tsuey-Yuan Huang, Feng-Chun Tsai

**Affiliations:** 1 Chang Gung University of Science and Technology, College of Nursing, Taiwan; 2 DaYeh University, College of Nursing and Sciences, Taiwan; 3 University of Kentucky, College of Nursing, USA; 4 Chang Gung University of Science and Technology, College of Nursing, Taiwan; 5 Chang Gung Memorial Hospital, Division of Cardiovascular Surgery, Taiwan

**Keywords:** Symptom trajectory, coronary artery bypass surgery, latent class growth model, fatigue, depression, angina

## Abstract

**Background:**

Coronary artery bypass grafting is one of the most common interventional revascularisation procedures used to treat coronary artery disease worldwide. With a wide variability in postoperative cardiac symptoms, identification of symptom trajectories during the 3-month postoperative recovery period may improve clinicians’ abilities to support symptom recovery.

**Aims:**

To identify distinct trajectories of cardiac symptoms seen over time in a cohort of patients during the 3-month post-coronary artery bypass grafting period, and determine clinical characteristics associated with different symptom trajectories postoperatively.

**Methods:**

A prospective trial used the cardiac symptom survey to determine patient symptoms at baseline prior to surgery, and at 1 week, 6 weeks and 3 months following coronary artery bypass grafting. A latent class growth model and multivariate logistic regression analyses were used.

**Results:**

Data were obtained from patients (*N*=198) undergoing coronary artery bypass grafting in six medical centres of Taiwan, through patient medical records and interviews. Based on their frequency, trajectories were explored for the six most common postoperative symptoms including angina, dyspnoea, fatigue, depression, sleep problems and anxiety. We identified two to three distinct classes of trajectories for each symptom. Age, longer intensive care unit stay, fewer vessels bypassed, off-pump coronary artery bypass grafting, smoking history and lack of regular exercise were associated with worse symptom outcome trends over time.

**Conclusions:**

Using this unique trajectories-based research method, we are able to achieve a better understanding of symptom recovery patterns over time among coronary artery bypass grafting patients. Recognising risk factors and potential recovery patterns prior to surgery may allow healthcare providers to deliver targeted discharge planning and individualised care after coronary artery bypass grafting.

## Introduction

Cardiovascular disease (CVD), and more specifically coronary artery disease is the major killer of American men and women, and causes about one of every six deaths in the United States.^1^ Over the past two decades in Taiwan, CVD has emerged as the second cause of death every year among adults. Experts have also noted a recent trend in Taiwan of an increased incidence of CVD among young adults aged 18–40 years.^2^ Thus, examining risks factors for CVD development and supporting patients in symptom recovery remains of utmost importance to cardiovascular nursing.

Coronary artery bypass grafting (CABG) is one of the most common interventional procedures used worldwide to treat coronary artery disease. Despite patient expectations of the immediate benefits of CABG, some symptoms (e.g. fatigue, surgical pain, shortness of breath and sleep problems) persist for weeks after hospital discharge, compromising the ability to return to a presurgery functional status.^3–6^ Symptom expression can also vary widely over time. Some symptoms can last a year or longer, adversely affecting quality of life.^7–9^

Discharge planning is routinely provided in hospitals to assist with post-CABG recovery. However, as hospital length of stay decreases and patient/nurse ratios substantially increase, less attention may be given to patient education and discharge planning. As a consequence, patients may be less well prepared to cope with adverse post-discharge symptoms. Moreover, symptom expression is driven by a variety of personal and clinical factors.^9^ While there is a large body of research on the symptom experience of patients undergoing CABG, there is limited information to guide patients to gauge their expected symptom trajectory during the critical 3-month post-surgery recovery period. A critical examination of symptom trajectories is important to guide patient education and nursing interventions during patient hospitalisation and post-discharge.

Given the dearth of research on symptom trajectories post-CABG, the aims of this study were to: identify different symptom trajectories over the immediate 3-month post-CABG recovery period, and to determine which patient demographic and clinical characteristics were associated with each symptom trajectory.

## Methods

### Study design

**Figure 1.**
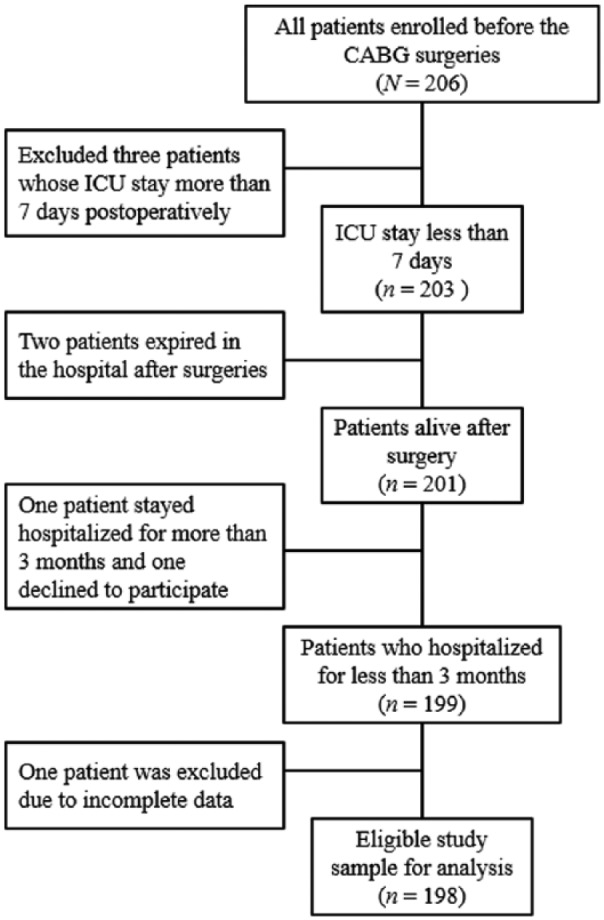
Flow chart of eligible patients.

A prospective study was conducted in which data were collected during assessments at baseline prior to surgery, and at 1 week, 6 weeks and 3 months following CABG. Patients waiting for their first CABG were eligible to participate if they were able to communicate in Mandarin or Taiwanese and had not been diagnosed with dementia or an active psychiatric illness. Patients who had CABG combined with valve replacement or who had organ failure, cancer, stroke or any physical condition requiring seven or more days in the intensive care unit (ICU) were also excluded. A sample of 198 adult patients undergoing CABG was recruited by referral from cardiac surgeons in six medical centres of Taiwan (Figure 1). Data were obtained from November 2010 to December 2011 through a review of the patients’ medical records and interviews.

### Measures

#### Personal and clinical characteristics

Based on the review of literature^10–12^ and clinical practice, the following clinical characteristics were obtained from the participants and from their medical records: type of CABG (on or off-pump), number of vessels bypassed, left ventricular ejection fraction, New York Heart Association (NYHA) functional classification, history of diabetes and hypertension and preoperative body mass index (BMI). Personal characteristics including gender, age, marital status, race, education, smoking history, exercise history and alcohol use were obtained from each participant at their baseline assessment.

#### The cardiac symptom survey

The cardiac symptom survey (CSS) was used to assess the symptom experience in individual patients. For this study, the Taiwan CSS was developed with permission from the original authors, using standard translation and back-translation procedures by the primary investigator. The Taiwan CSS was further validated by two English-speaking researchers and a bilingual Taiwanese layperson. The original CSS was first developed and tested in 2001 on 35 post-CABG patients over the first 6 weeks post-surgery recovery period.^6^ Further content validity and responsiveness testing was conducted on a sample of 90 post-CABG patients.^13^

The final CSS is a 40-item scale based on the symptoms management model and is used to assess patients’ perceptions, evaluations and responses to 10 symptoms commonly experienced post-CABG.^13^ First, patients rate their perception of having one of 10 symptoms (angina, shortness of breath, fatigue, depression, trouble sleeping, pain from surgery, swollen legs, palpitation, anxiety and poor appetite) on a scale of 0 = no and 1 = yes. Then, if a symptom is present, patients rate their evaluation of symptoms by scoring the frequency and severity of the symptoms on separate scales from 0 to 10, with a rating of 10 indicating the highest frequency or severity. Similarly, the patient can respond to symptoms with respect to interference with their physical activity and enjoyment of life on the same 0 to 10 scale; however, for the purpose of this study, response to physical activity and enjoyment of life were not included in calculating severity scores. Thus the evaluation of the symptoms was estimated by calculating a mean score of frequency and severity ratings.

### Procedure

The study was approved by the institutional review boards at all six hospitals. Patients provided signed informed consent prior to data collection. Demographic and clinical data and the responses to the baseline CSS were collected by a trained research nurse prior to surgery. The 1-week post-CABG CSS was completed during the follow-up clinic visit or in the hospital if the patient had not been discharged. The CSS at the 6-week and 3-month time points was completed during the follow-up clinic visits. If needed, a trained research nurse helped the patient complete the instrument. Data collecting processes were systematically managed and closely monitored throughout the whole study. There were a very few occasions that the follow-up interviews were not available so CSS questionnaires were mailed to participants and then answered by phone interview at a requested time by the patient. Data were then analysed by an independent statistician after data collection.

### Data analysis

The surveys were completed in full by participants with no missing data. Data were inputted into a data analysis software by trained research assistants. Standard data checking procedures, such as random check of transferred data, were performed to ensure the accuracy of inputted data.

Demographic and clinical data were described using means with standard deviations for interval/ratio data and frequencies with percentages for nominal and ordinal data. Means with standard deviations were also used to describe symptom perception scores from pre-CABG to 3-month time points. To describe better the individual trajectory course of the study variables over time, the latent class growth model (LCGM), which assumed each patient is composed of a mixture of groups with distinctive trajectories, was used. LCGM was used to identify whether underlying patterns or ‘latent classes’ existed in the trajectories of patients’ symptoms. To determine the optimal number of trajectories, the Bayesian information criterion (BIC) was used, with a lower BIC value indicating a better fit.^14^ Prior to these statistical analyses, patients’ characteristics including age, gender, BMI, ICU stay, history of diabetes, history of hypertension, number of vessels bypassed, CABG type, work status, smoking history, alcohol use and regular exercise activity were re-coded into dichotomous variables.

For bivariate comparisons between or among classes delineated from LCGM, chi-square tests were used for all discrete variables. To decrease the possibility of making a type I error, we adjusted the alpha level using the Bonferroni convention with six variables. In order to determine which variables were independently associated with symptom trajectory classes, multivariable binary logistic regression (or multivariable multinomial logistic regression) was performed. All data were analysed using SPSS for windows version 15.0 and SAS 8.2.

## Results

### Demographic and clinical characteristics of the sample

The personal and clinical characteristics of the sample are presented in Table 1. Most participants were men (81%) with a mean age of 61.1 years. Participants were mostly educated at a high school or greater level (63%). More than half of participants smoked cigarettes or used alcohol and did not regularly exercise; and more than two-thirds had heart failure and hypertension. Half of the participants had an ‘off pump’ CABG procedure and the mean length of ICU stay was 3.6 days.

**Table 1.** Sample and clinical characteristics at baseline (*N*=198).

Characteristics	Number (%)	Mean±SD (range)
**Sociodemographic**		
Gender		
Male	161 (81%)	
Female	37 (19%)	
Age, years		61.1 ± 11.1 (23–87)
<65 years	124 (63%)	
≧65 years	74 (37%)	
Education level		
Elementary or below	73 (37%)	
High school	92 (46%)	
College or above	33 (17%)	
Work status		
Employed	100 (51%)	
Unemployed	98 (49%)	
Body mass index, kg/m^2^		25.6 ± 3.4 (19.7–40.2)
Non-obese (<27)	138 (70%)	
Obese (≧27)	60 (30%)	
**Lifestyle**		
Tobacco use	87 (44%)	
Alcohol use	44 (22%)	
Not regular exercise	106 (54%)	
**Comorbidities**		
Hypertension	155 (78%)	
Diabetes mellitus	106 (54%)	
Heart failure (NYHA ≧1)	128 (65%)	
**Clinical characteristics**		
Pre-op LVEF, %		53.8 ± 13.7 (16–87)
≧50%	133 (67%)	
<50%	65 (33%)	
Vessel bypassed		
LMA	3 (2%)	
RCA	158 (80%)	
LAD	196 (99%)	
Lcx	169 (85%)	
Number of vessel bypassed		2.7 ± 0.6 (1–4)
≧3	142 (72%)	
≦2	56 (28%)	
Type of CABG		
On pump, arrest	53 (27%)	
On pump, beating	38 (19%)	
Off pump	107 (54%)	
Length of ICU stay, days		3.6 ± 1.5 (1–7)
<4 days	102 (52%)	
≧4 days	96 (48%)	

LMA: left main coronary artery; RCA: right coronary artery; LAD: left anterior descending artery; Lcx: left circumflex artery; NYHA: New York Heart Association; LVEF: left ventricular ejection fraction; CABG: coronary artery bypass grafting; ICU: intensive care unit.

### Trajectories of symptoms

**Figure 2.**
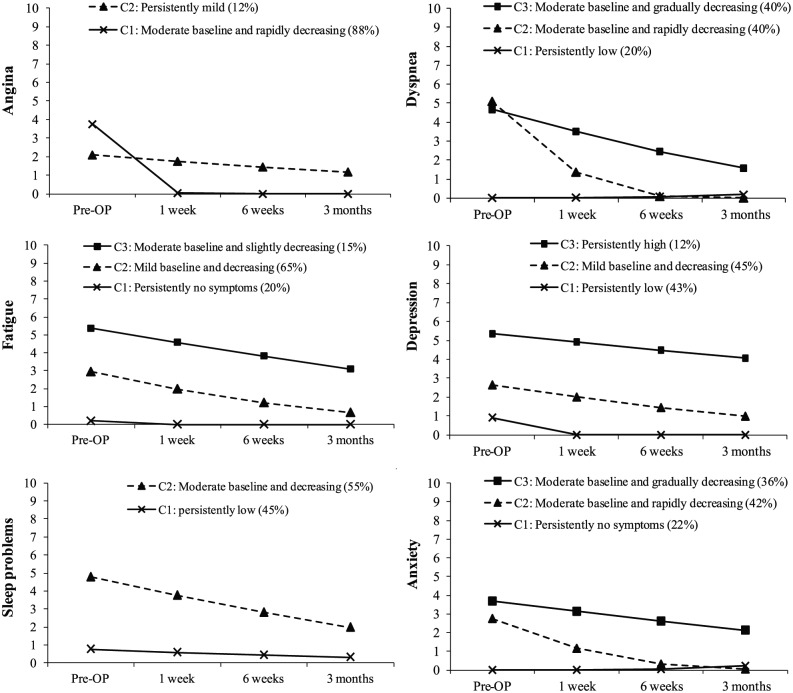
Observed trajectories of each symptom.

All symptoms improved consistently in the postoperative period with the exception of surgical pain and lower extremity oedema (Table 2). Only six out of 10 symptoms (i.e. angina, dyspnoea, fatigue, depression, sleep problems and anxiety) were chosen for further LCGM analyses because they had average scores of 2 or more. Figure 2 visually depicts the observed trajectories of these six symptoms.

**Table 2.** Symptom perception scores (mean±SD) for each symptom throughout the study period.

Symptom	Pre-op	1 Week	6 Weeks	3 Months
Angina	3.71 ± 3.01	0.19 ± 0.81	0.23 ± 0.98	0.18 ± 0.84
Dyspnoea	4.13 ± 3.31	1.66 ± 1.88	1.11 ± 1.85	0.69 ± 1.28
Fatigue	3.08 ± 3.02	1.79 ± 2.10	1.37 ± 1.85	0.97 ± 1.59
Depression	2.42 ± 2.64	1.41 ± 2.00	1.24 ± 1.86	0.96 ± 1.70
Sleep problems	2.99 ± 3.35	2.33 ± 2.37	1.77 ± 2.27	1.16 ± 1.86
Surgical pain	0.24 ± 1.10	3.20 ± 2.01	2.04 ± 1.71	1.04 ± 1.53
Swollen legs	0.84 ± 2.23	1.11 ± 1.74	0.78 ± 1.46	0.66 ± 1.46
Palpitation	1.66 ± 2.63	0.48 ± 1.22	0.28 ± 0.94	0.24 ± 0.80
Anxiety	2.77 ± 2.78	1.41 ± 1.79	1.10 ± 1.68	0.90 ± 1.56
Poor appetite	1.03 ± 2.05	1.16 ± 2.12	0.61 ± 1.54	0.34 ± 1.06

OP: operation; higher scores indicate greater symptom burden.

There were two distinct classes in the symptom expression of angina and sleep problems. Among patients with angina, the first trajectory, ‘moderate baseline and rapidly decreasing angina’, had 88% of patients with a trajectory characterised by a higher level of angina before surgery but a significant decrease at follow-up; and the second trajectory, ‘persistently mild angina’ with 12% of patients, demonstrated a trajectory with only a small (non-significant) reduction from a low baseline level of angina. In a similar fashion, among those with sleep problems, the first trajectory, ‘persistently low sleep problem’, consisted of 45% of patients with very mild sleep problems before CABG that remained stable after surgery; and the second trajectory, ‘moderate baseline and decreasing sleep problems’, comprised 55% of patients with a higher level of sleep problems at baseline with a significant decrease over time.

Moreover, there were three separate classes each for symptom expressions of dyspnoea, fatigue, depression and anxiety. Among those with dyspnoea, trajectory 1 (persistently low dyspnoea) consisted of 20% of patients with no/low dyspnoea symptoms before CABG and very low-grade breathlessness at the follow-up assessment, trajectory 2 (moderate baseline and rapidly decreasing dyspnoea) had 40% of patients with the highest scores of breathlessness before surgery and a significant decrease over time, and trajectory 3 (moderate baseline and gradually decreasing dyspnoea) had 40% of patients who had breathlessness before and after CABG that gradually improved, but did not completely disappear. Similar patterns of trajectories were evidenced for fatigue, with trajectory 1 (persistently no fatigue symptoms) comprising 20% of patients, trajectory 2 (mild baseline and decreasing fatigue symptoms) consisting of 65% of patients, and trajectory 3 (moderate baseline and slightly decreasing fatigue symptoms) representing 15% of patients. Among those with depression, trajectory 1 (persistently low depression) comprised 43% of patients, trajectory 2 (mild baseline and decreasing depression) was composed of 45% of patients, and trajectory 3 (persistently high depression) included 12% of patients. Finally, among those with anxiety symptoms, trajectory 1 (persistently no anxiety symptoms) included 22% of patients, trajectory 2 (moderate baseline and rapidly decreasing anxiety) comprised 42% of patients, and trajectory 3 (moderate baseline and gradually decreasing anxiety) included 36% of patients.

### Factors associated with symptom trajectories

Table 3 displays the results of bivariate analysis of demographic and clinical variables by symptom classes. There were no differences in demographic or clinical characteristics among the trajectories for angina and anxiety. In dyspnoea trajectories, older age and longer length of ICU stay were associated with the worst symptom trajectory. Among patients experiencing fatigue, longer ICU stay was associated with the worst trajectory. Among those with depression, longer ICU stay, being unemployed, and no regular exercise prior to surgery were associated with a worse trajectory. Finally, for sleep problems, the fewer vessels bypassed, the worse the symptom trajectory.

**Table 3.** Percentage of patients in each symptom trajectory based on sociodemographic and clinical characteristics.

Variable	Angina		Dyspnoea			Fatigue			Depression			Sleeping		Anxiety		
	C1	C2	C1	C2	C3	C1	C2	C3	C1	C2	C3	C1	C2	C1	C2	G3
	(*n*=175)	(*n*=23)	(*n*=39)	(*n*=79)	(*n*=80)	(*n*=40)	(*n*=128)	(*n*=30)	(*n*=85)	(*n*=89)	(*n*=24)	(*n*=89)	(*n*=109)	(*n*=43)	(*n*=84)	(*n*=71)
Age ≧65	36	48	***18***	***35***	***49***	30	36	53	32	38	54	31	42	33	37	41
Female gender	18	26	13	20	20	13	17	33	16	19	25	16	21	7	23	21
Obese (BMI ≧27)	30	30	28	32	30	38	27	37	32	30	25	34	28	33	27	32
ICU stay ≧4 days	45	74	***46***	***35***	***63***	***35***	***45***	***83***	***40***	***52***	***67***	45	51	47	45	54
Diabetes	55	43	54	58	49	48	55	53	47	58	58	47	59	44	55	58
Hypertension	79	74	74	76	83	78	78	80	78	79	79	76	80	74	79	80
Vessels bypassed ≦2	28	30	13	32	33	18	28	43	25	27	46	***18***	***37***	14	35	30
On pump	49	26	33	48	50	40	48	47	49	44	42	45	47	51	43	46
Unemployment	48	61	44	44	58	48	48	60	***41***	***52***	***71***	39	58	49	42	59
Tobacco use	43	48	44	39	49	33	48	43	36	48	54	38	49	40	36	56
Alcohol use	22	22	26	22	21	28	22	17	24	25	8	30	16	28	19	23
No regular exercise	51	74	51	51	58	45	56	53	***42***	***63***	***58***	55	52	51	46	63

The number in the cell indicates the percentage of patients.

BMI: body mass index; ICU: intensive care unit; LVEF: left ventricular ejection fraction; C: class.

Numbers in bold italic represent a significant chi-square.

The results of the multivariate logistic regression analyses are presented in Table 4. For these regression analyses we used class 1 in each of the six symptom trajectories as the reference group because across classes and symptom trajectories, patients in class 1 had an overall better outcome. Patients who had a longer ICU stay (odds ratio (OR)_2/1_ 5.03, *P*=0.008) were significantly more likely to be in the worse angina trajectory. On the other hand, those patients who had on-pump CABG (OR_2/1_ 0.18, *P*=0.0085) were more likely to make a quicker recovery in terms of angina symptoms. Patients who were older than 65 years (OR_3/1_ 10.78, *P*=0.001) were significantly more likely to have the worst dyspnoea trajectory. Patients who had a longer ICU stay (OR_3/1_ 8.06, *P*=0.003) or fewer vessels bypassed (OR_3/1_ 6.28, *P*=0.006) were significantly more likely to have the worst fatigue trajectory.

**Table 4.** Factors associated with trajectory of each symptom.

Variable	Angina		Dyspnoea				Fatigue				Depression				Sleeping		Anxiety			
	OR_2/1_	*P*	OR_2/1_	*P*	OR_3/1_	*P*	OR_2/1_	*P*	OR_3/1_	*P*	OR_2/1_	*P*	OR_3/1_	*P*	OR_2/1_	*P*	OR_2/1_	*P*	OR_3/1_	*P*
Age ≧65	1.57	NS	4.86	NS	***10.78***	***0.001***	2.45	NS	4.47	NS	1.61	NS	4.21	NS	1.57	NS	1.03	NS	1.75	NS
Female gender	2.08	NS	1.23	NS	1.13	NS	1.18	NS	3.24	NS	1.33	NS	2.05	NS	1.50	NS	3.27	NS	5.06	NS
Obese (BMI ≧27)	1.48	NS	1.33	NS	1.43	NS	0.60	NS	2.14	NS	0.86	NS	0.98	NS	0.82	NS	0.64	NS	0.89	NS
ICU stay ≧4 days	***5.03***	***0.008***	0.27	NS	1.04	NS	1.20	NS	***8.06***	***0.003***	1.92	NS	3.65	NS	1.36	NS	0.59	NS	0.99	NS
Diabetes	0.47	NS	1.27	NS	0.68	NS	1.45	NS	1.55	NS	1.71	NS	1.89	NS	1.97	NS	1.72	NS	1.58	NS
Hypertension	0.56	NS	1.11	NS	1.94	NS	0.90	NS	1.02	NS	0.86	NS	0.71	NS	0.95	NS	1.14	NS	1.24	NS
Vessels bypassed ≦2	0.81	NS	4.77	NS	4.87	NS	2.37	NS	***6.28***	***0.006***	1.21	NS	3.25	NS	***3.36***	***0.001***	***4.85***	***0.004***	3.73	NS
On pump	***0.18***	***0.008***	3.65	NS	3.22	NS	1.13	NS	0.67	NS	0.54	NS	0.51	NS	0.86	NS	0.64	NS	0.61	NS
Unemployment	1.68	NS	0.87	NS	1.22	NS	0.77	NS	1.50	NS	1.51	NS	2.62	NS	1.81	NS	0.72	NS	1.58	NS
Tobacco use	1.93	NS	1.16	NS	2.57	NS	2.72	NS	4.99	NS	2.16	NS	***7.23***	***0.002***	2.56	NS	0.98	NS	3.06	NS
Alcohol use	0.91	NS	0.66	NS	0.62	NS	0.61	NS	0.51	NS	0.93	NS	0.18	NS	0.37	NS	0.57	NS	0.69	NS
No regular exercise	3.09	NS	1.59	NS	2.66	NS	2.39	NS	1.80	NS	***2.67***	***0.005***	2.88	NS	0.95	NS	0.80	NS	1.73	NS

OR: odds ratio; NS: not statistically significant; OR_2/1_: class 2 relative to class 1; OR_3/1_: class 3 relative to class 1; BMI: body mass index; ICU: intensive care unit; LVEF: left ventricular ejection fraction; C: class.

Numbers in bold italic represent a significant odds ratio.

In terms of recovery from depressive symptoms, patients who smoked were significantly more likely to have the worst trajectory (OR_3/1_ 7.23, *P*=0.002), while patients who did no regular exercise (OR_2/1_ 2.67, *P*=0.005) were more likely to experience slower depression symptom resolution. Patients who had had fewer vessels bypassed (OR_2/1_ 3.36, *P*=0.001) were more likely to have a poor sleep problem trajectory. Finally, patients who had fewer vessels bypassed were significantly more likely to have a slow recovery from symptoms of fatigue (OR_2/1_ 3.36, *P*=0.001) and anxiety (OR_2/1_ 4.85, *P*=0.004).

## Discussion

Recovery from cardiac surgery is a dynamic process that involves interconnections between physical, psychological and social health and wellbeing. We found multiple distinct symptom trajectories for the six most disturbing symptoms after CABG: angina, dyspnoea, depression, fatigue, sleep problems and anxiety. Several characteristics including age, longer ICU stay, fewer vessels bypassed, off-pump CABG, smoking history and lack of regular exercise are associated with worse symptom trajectories in patients following CABG surgeries at a 3-month period. Recognising these risk factors and corresponding potential recovery patterns prior to surgery may allow healthcare providers to deliver targeted discharge planning and more individualised care for patients after CABG.

Symptom trajectories have been commonly described in cancer patients during and after treatment.^15–17^ By identifying unique trajectories for a variety of symptoms, cancer research in this area has resulted in patient-centred care and interventions to reduce symptom burden (e.g. depressive symptoms, headache, pain and fatigue), thereby improving quality of life.

More recently, symptom trajectories have been described in cardiac patients with possible acute coronary syndrome (ACS).^18^ In a study by Knight et al. (2016),^18^ the trajectories of symptom severity for eight cardiac symptoms were measured at 1 month and 6 months after an emergency department visit for potential ACS. This study proposed that the identification of symptom trajectories could help clinicians tailor care to specific individuals. Thus, although ours is the first study to define symptom trajectories in CABG patients using LCGM, evidence from other conditions suggests that using information about symptom trajectories may lead to more targeted and effective interventions for CABG symptoms.

In addition to identifying symptom trajectories, we investigated demographic and clinical characteristics that might predict membership in specific trajectories for each symptom. We found that patients who had a longer ICU stay (≧4 days) were more likely to have a ‘persistently mild’ level of angina that did not resolve after surgery than those with shorter ICU stays. Prior to our study, no specific link between a longer ICU stay and a worse angina symptom recovery had been identified. However, a study by Ghotkar and colleagues^19^ suggested that angina was a risk factor for prolonged ICU stay. This is likely to be because patients who have more severe angina usually have greater myocardial damage than those with less severe angina. This may result in a longer cardiac recovery process in terms of the balance of oxygen demand and supply.

We additionally found that the use of traditional on-pump CABG (compared to off-pump CABG) was related to a trajectory characterised by a rapid reduction of angina after surgery. Unfortunately, few studies have examined recurrent angina in relation to on-pump and off-pump procedures. For example, van Dijk and colleagues (2001) conducted a multicentre randomised control trial and reported a similar rate of recurrent angina for both off-pump and on-pump groups (4.9% vs. 4.3%) at 1 month of follow-up.^20^ Furthermore, in a meta-analysis of randomised controlled trials, Kowalewski et al. (2016) reported that there was no difference between off-pump and on-pump procedures in terms of myocardial infarction (MI) within 30 days after surgery; however, the benefits of on-pump CABG were greater in high-risk patients with MI.^10^ Yet, a meta-analysis of randomised controlled trials (16,904 patients from 51 studies) reported no significant differences in major adverse cardiac and cerebrovascular events between on-pump versus off-pump procedures, but a higher mid-term graft failure and need for revascularisation were associated with off-pump CABG.^12^ These study findings may support our current finding that the on-pump group had a better recovery of angina after surgery.

We found that patients who were 65 years or older were more likely to have the worst dyspnoea trajectory. Despite the fact that we found age to be a significant factor associated with the dyspnoea trajectory, a previous study of 268 patients demonstrated that, in general, cardiac-related symptoms 6 months after CABG did not vary by age.^21^ Those investigators, however, only considered average symptom ratings across the group, and did not identify symptom trajectories, which always demonstrate greater heterogeneity of response. Therefore, more studies are needed to determine the effects of age on dyspnoea trajectories.

We also found that, compared to patients who had three or more vessels bypassed, those who had one or two vessels bypassed were significantly more likely to experience the worst fatigue symptom trajectory which did not fully resolve at 3 months. This finding is important because fatigue after CABG is a prominent symptom and fatigue is a predictor of both poor overall prognosis and mortality among heart failure patients.^22^ It is also important to note that up to 15% of the patients in our study had only a slight reductions in their fatigue symptoms postoperatively. We postulate that because patients with one and two-vessel disease who undergo CABG usually have blockages at the left main and/or proximal left anterior descending coronary artery, they often require a longer vessel clamp time during surgery. Due to the increased length of their CABG procedure and the associated potential for longer ICU stay, patients with fewer vessels bypassed may be more likely to experience a longer recovery. Hence, future studies may examine the severity associated with the numbers of vessels bypassed in relation to fatigue symptoms. For example, an examination of the trajectory of fatigue among those with left and/or proximal left anterior descending coronary artery may enhance our understanding of how to help such patients with symptom recovery post-CABG.

Like fatigue, depression is another prominent negative symptom that patients in our study experienced after CABG. Depression may be a predictor of lack of improvement in a patient’s physical health after CABG surgery.^23^ We found that the majority (88%) of participants had trajectories demonstrating significant decreases in their depression level over time. These results are congruent with previous studies that demonstrated that patients’ levels of depression and anxiety decreased significantly from before to after surgery.^11,24^ Therefore, continuing to address depression post-CABG remains an area of clinical importance.

Smoking was associated with the worst depression trajectory. We further demonstrated that patients who engaged in substantial physical activity had the best depression trajectory. These trajectories are consistent with data showing that lifestyle behaviours are strongly associated with the expression of depressive symptoms.^25^ Therefore, encouraging smoking cessation post-CABG is highly recommended as a means to reduce symptom burden.

We also found that patients with fewer vessels bypassed were more likely to have worse outcomes with sleep disturbance and anxiety symptom trajectories. Indeed, as we asserted earlier, when patients have fewer vessels bypassed, they are more likely to have on-pump CABG, which is often associated with a longer ischaemic time during the surgery, may involve a more complex and difficult recovery and can in turn result in more difficulties in sleeping during the recovery period. The association between few vessels bypassed and the anxiety trajectory is a novel finding and additional research may be needed to explain this result further. For example, assessing anxiety levels using standardised instruments (e.g. the brief symptom inventory or generalised anxiety disorder scale) among patients with differing numbers of vessels bypassed may shed more light on this phenomenon

### Strengths and limitations

There are several strengths of the current study. The sample was highly representative of patients treated at the medical centres. Nearly 98% of patients approached agreed to participate in the study. This may be, in part, due to cultural differences. In Taiwan, the majority of patients trust their doctors and healthcare providers so there are fewer obstacles to enrolling patients in the study. Another strength of this study is its prospective design, with repeated measurement of symptoms over time, which enables us to examine the course of symptoms with an objective outcome. Although we could have used a mixed model analysis, the strength of LCGM is that it categorises individual risk trajectories into homogenous subgroups whereas mixed models rely on a specific framework or theories to derive such subgroups.^14^

On the other hand, this current study has limitations. First, the composite scores of some symptoms were relatively low, which might result in less reliable estimates of symptom trajectories. Second, given the nature of the analysis method used in this study, we can only speculate as to the reasons why patient clinical trajectories are associated with certain characteristics. Third, we were not fully able to determine how surgical complications may have affected our main outcomes. However, three (1.5%) patients required a second surgery to stop bleeding after the original CABG, one (0.5%) patient required haemodialysis for renal failure, three (1.5%) patients had sternal wound infections requiring operation, and two (1.0%) patients were re-admitted to the ICU. Hence, these complications were not considered in the analysis because of the low incidences. Finally, given that the data were derived from a sample from Taiwan, it is possible that the findings may not be generalisable beyond the study setting. The experiences of several of the symptoms endorsed by participants may be nuanced with cultural norms unique to the Chinese culture in Taiwan. However, given these limitations, future studies are required to investigate this topic further.

## Conclusion

We have contributed to this body of literature by using LCGM to define distinct trajectories of each symptom from preoperative to 1-week, 6-week and 3-month periods after surgery. We identified several symptom trajectories in which there were groups of patients whose symptoms did not resolve during the study period or only decreased slightly. These findings are important because they demonstrate that the symptom experience after CABG is not homogenous and that not all patients experience resolution of symptoms at the same rate, if at all. This study allows healthcare providers and patients to understand and anticipate the course of CABG recovery based on individual patient characteristics.

Implications for clinical practice from the study include: patients who are 65 years or older could be encouraged to do more breathing exercises to prevent worse dyspnoea after surgery; patients who have a longer length of ICU stay (⩾4 days) should be closely monitored for the development of worse angina and fatigue postoperatively; patients who have one or two vessels bypassed should be instructed on interventions to prevent fatigue and sleep problems, as well as relaxation techniques to reduce anxiety; patients undergoing off-pump CABG should be able to recognise signs and symptoms of angina for a higher graft failure rate; and patients who smoke and do not regularly exercise should be screened for depression. These recommendations for clinical practice can be instrumental in guiding more informed assessments and targeted treatment of patients post-CABG. Ultimately, this advanced assessment can aid in minimising the burden of symptoms for CABG patients.
